# Strengthening community-based surveillance: lessons learned from the 2018–2020 Democratic Republic of Congo (DRC) Ebola outbreak

**DOI:** 10.1186/s13031-023-00536-7

**Published:** 2023-08-30

**Authors:** Jennifer OKeeffe, Emi Takahashi, John Otokoye Otshudiema, Emile Malembi, Célestin Ndaliko, Nathanaël Mutimatonda Munihire, Grazia Caleo, Antonio Isidro Carrion Martin

**Affiliations:** 1 Médecins Sans Frontières, Goma, Democratic Republic of Congo; 2 World Health Organization, Kinshasa, Democratic Republic of Congo; 3 Minister of Public Health, Kinshasa, Democratic Republic of Congo; 4 Médecins Sans Frontières, London, UK; 5 Norwegian Red Cross, Oslo, Norway

**Keywords:** Community based surveillance, Ebola, Outbreak, Democratic Republic of Congo

## Abstract

**Introduction:**

There has been little documentation of the large networks of community health workers that contributed to Ebola Virus Disease (EVD) surveillance during the 2018–2020 Democratic Republic of Congo (DRC) epidemic in the form of community-based surveillance (CBS). These networks, comprised entirely of local community members, were a critical and mostly unrecognized factor in ending the epidemic. Challenges with collection, compilation, and analysis of CBS data have made their contribution difficult to quantify. From November 2019 to March 2020, the DRC Ministry of Health (MoH), the World Health Organization (WHO), and Médecins Sans Frontières (MSF) worked with communities to strengthen existing EVD CBS in two key health areas in Ituri Province, DRC. We describe CBS strengthening activities, detail collaboration with communities and present results of these efforts. We also provide lessons learned to inform future outbreak responses.

**Methods:**

As the foundation of CBS, community health workers (CHW) completed training to identify and report patients who met the EVD alert definitions. Alerts were investigated and if validated, the patient was sent for isolation and EVD testing. Community members provided early and ongoing input to the CBS system. We established a predefined ratio of community- elected CHW, allocated by population, to assure equal and adequate coverage across areas. Strong performing CHW or local leaders managed the CHWs, providing a robust supervision structure. We made additional efforts to integrate rural villages, revised tools to lighten the reporting burden and focused analysis on key indicators. Phased roll-out of activities ensured time for community discussion and approval. An integrated treatment center (ITC) combined EVD testing and isolation with free primary health care (PHC), referral services, and an ambulance network.

**Results:**

A total of 247 CHW and supervisors completed training. CBS had a retention rate of 94.3% (n = 233) with an average daily reporting rate of 97.4% (range 75.0-100.0%). Local chiefs and community leaders participated in activities from the early stages. Community feedback, including recommendations to add additional CHW, run separate meetings in rural villages, and strengthen PHC services, improved system coverage and performance. Of 6,711 community referrals made, 98.1% (n = 6,583) were classified as alerts. Of the alerts, 97.4% (n = 6,410) were investigated and 3.0% (n = 190) were validated. Of the community referrals, 73.1% (n = 4,905) arrived for care at the ITC. The contribution of CBS to total alerts in the surveillance system increased from an average of 47.3% in the four weeks prior to system strengthening to 69.0% after. In one of the two health areas, insufficient reporting in rural villages suggested inadequate coverage, with 8.3% of the total population contributing 6.1% of alerts.

**Discussion:**

CBS demonstrated the capacity of community networks to improve early disease detection and expand access to healthcare. Early and consistent community involvement proved vital to CBS, as measured by system performance, local acceptance of EVD activities, and health service provision. The CBS system had high reporting rates, number of alerts signaled, proportion of alerts investigated, and proportion of community referrals that arrived for care. The change in contribution of CBS to total alerts may have been due in part to system strengthening, but also to the expansion in the EVD suspect case definition. Provision of PHC, referral services, and an ambulance network linked EVD response activities to the existing health system and facilitated CBS performance. More importantly, these activities provided a continuum of care that addressed community prioritized health needs. The involvement of local health promotion teams was vital to the CBS and other EVD and PHC activities. Lessons learned include the importance of early and consistent community involvement in surveillance activities and the recommendation to assure local representation in leadership positions.

## Background

The 2018 Ebola virus disease (EVD) outbreak in the Democratic Republic of Congo (DRC) began on August 1, 2018 [1]. In July 2019, the World Health Organization (WHO) declared the outbreak a public health emergency of international concern (PHEIC) [2]. The outbreak was the largest in the DRC and the second largest in history after the 2014–2016 West African epidemic. In the DRC, there were over 3,470 cases (3,317 confirmed, 153 probable) and 2,287 deaths, with a global case fatality rate (CFR) of 65.9% [3]. Insecurity in Eastern DRC posed challenges for the EVD response with difficulty accessing affected areas [4], frequent attacks on healthcare [5], and campaigns of misinformation [6]. The mobile population created challenges for contact tracing, testing, and isolation [7]. The response was characterized by poor community engagement and a weak early warning system that missed suspect cases and community deaths [8]. This resulted in delayed access to care, late alerts, and missed opportunities for outbreak control [9].

Community health workers (CHW) dedicating time in the form of community-based surveillance (CBS) have been used in low-income and fragile contexts to supplement facility-based surveillance [10]. Their efforts have been shown to improve case detection, reduce the delay of disease signals, and provide surveillance coverage in hard-to-reach areas [11]. In addition, they are effective in collecting health information outside the formal health system or for stigmatized diseases [12]. CHW were a valuable resource in ending the 2014–2016 West African EVD epidemic in Guinea [13], Sierra Leone [14], and Liberia [15].

In Eastern DRC, Mambasa health zone in Ituri Province is a major rural trading stop, with an approximate population of 147,000. Most EVD transmission occurred between late June and October 2019 with the last cases recorded in January 2020 [9]. By the end of the outbreak, Mambasa had registered 83 confirmed and probable EVD cases with 21 deaths (CFR: 25.3%). The national EVD response, known by its French title the “Riposte”, was a collaboration under the lead of the DRC Ministry of Health (MoH) with WHO acting as technical advisor. From October (week 43) to mid-November (week 48, inclusive) 2019, the Riposte operated a CBS system across Mambasa health zone. This period is subsequently referred to as “pre-system strengthening”. In November 2019, Médecins Sans Frontières Operational Center Amsterdam (MSF) partnered with the Riposte. Together with the communities, we strengthened the existing CBS in two health areas in the zone, Binase and Salama. The Binase CBS system was operational for 17 weeks, beginning in November (week 49) 2019, subsequently referred to in this paper as “post-system strengthening”. The Salama CBS system was operational for 7 weeks, beginning in February (week 6) 2020. Activities ended in March (week 13 inclusive) 2020, after 42 days, two EVD incubation periods, without transmission.

Challenges with collection, compilation, and analysis of CBS data have made the results of CHW efforts difficult to quantify. This case study describes the strengthening activities put in place and presents selected results which represent the contributions of CHW to the EVD surveillance under CBS. We highlight the challenges, successes, lessons learnt and recommendations, to help inform future responses.

## Methods

### CBS system

An indicator-based CBS [12], where CHWs conduct systematic collection of structured data, was implemented as part of EVD surveillance activities [16]. The DRC MoH has an established protocol for CBS based on WHO guidelines [17]. The Riposte adapted these for use in the EVD outbreak employing a cohort of CHW who performed routine household visits. CHW also sensitized community members on EVD prevention and control and encouraged them to seek care if they were sick. During household visits, CHW identified sick persons and raised an alert if a person met the EVD alert definition of someone potentially infected with Ebola virus disease. In January 2020, after a new cluster of cases occurred in the health zone, the alert and suspect case definitions were expanded to be more sensitive. These changes were made across the entire surveillance system. The new definition considered all sick persons as EVD alerts (Table 1. EVD Case Definitions).

**Table 1:** EVD Case Definitions

	Original Case Definitions (Before January 2020)	Expanded Case Definitions (After January 2020)
**Alert Definitions**	Fever that does not respond to treatment	Any symptom or illness
	Unexplained bleeding	Unexplained bleeding or miscarriage
	Sudden unexplained death	Any death
**Suspect Case Definitions**	Fever + contact	Any symptom + contact
	Fever + presence in active zone in past 21 days	Fever + presence in active zone in past 21 days
	Fever + 3 EVD associated symptoms	3 EVD associated symptoms
	Unexplained bleeding	Unexplained bleeding or miscarriage
	Sudden unexplained death	Any death

**Fig. 1:**
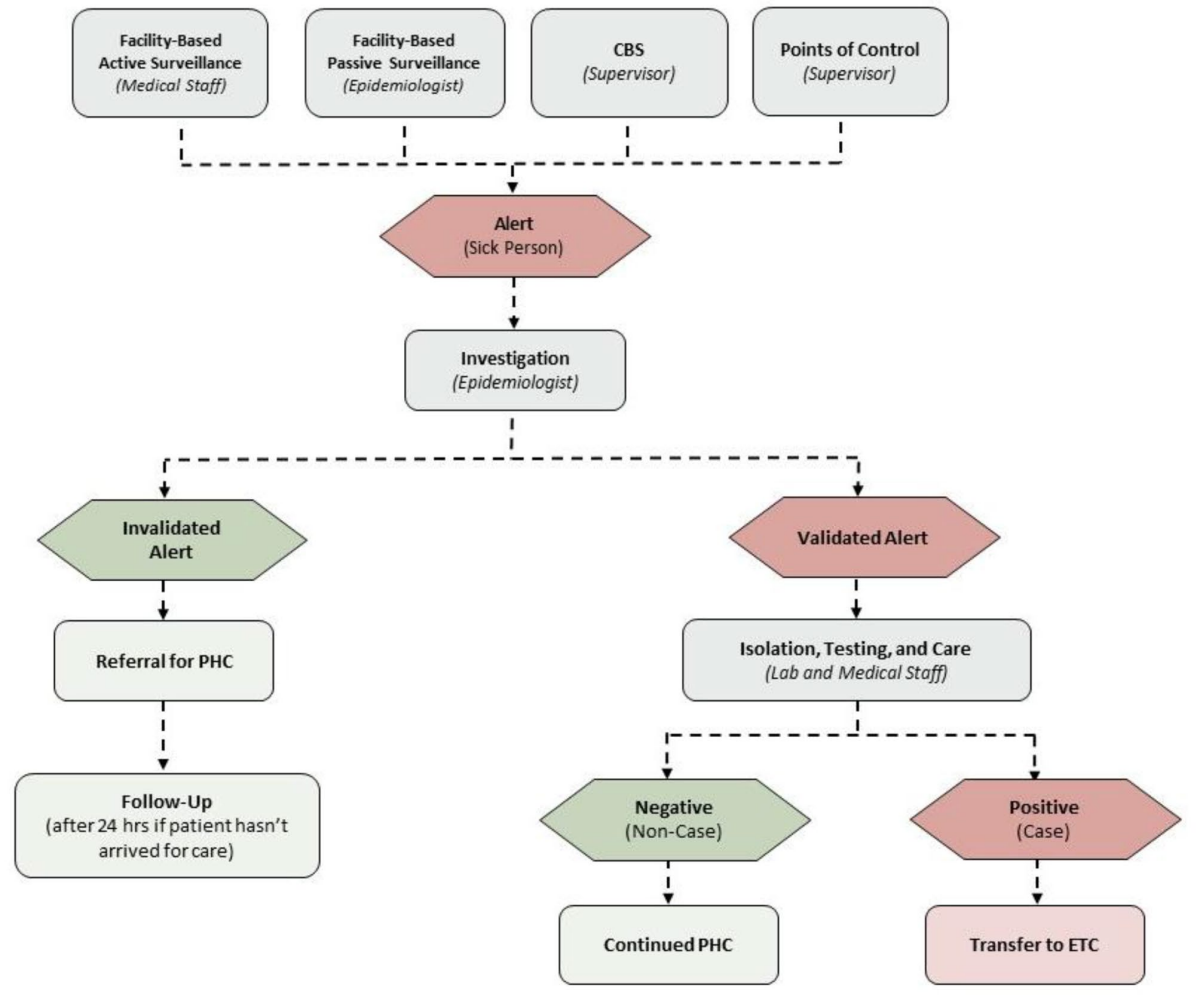
EVD Surveillance Flow Diagram

Supervisors collected alerts reported by CHW each day. Epidemiologists or clinical staff investigated the alerts to validate whether they fit the suspect case definition (Fig. 1. EVD Surveillance Flow Diagram). If an alert was validated as a suspect case, the patient was admitted to an integrated treatment center (ITC) for isolation and testing. Invalidated alerts were referred to the ITC for primary health care (PHC). Investigators responded to alerts within 24 h. In addition to daily reports, supervisors compiled the alerts in the form of referral slips. One copy of the referral slip was given to the patient, and another given to the surveillance team who deposited the slips at the ITC triage.

Originally, if there was an alert, investigators would visit the patient at home. After the expanded case definition, the number of alerts prevented investigation at home due to the burden of travel time and logistical constraints. Instead, patients were referred to the ITC for investigation. If they failed to arrive at the ITC after 24 h, investigators would travel to their residence to do the alert investigation. Patient arrival was not tracked in the reporting system. Rather, when patients arrived for care, the triage nurse would match the patient slip with the collection of slips delivered daily by the surveillance team. Each morning, surveillance team members would check with triage for unmatched referral slips and visit those households to conduct case investigations.

CBS supplemented active and passive facility-based surveillance, and point-of-control surveillance. In active facility-based surveillance, staff visited health facilities, pharmacies, and traditional healers to identify suspect cases. In passive facility-based surveillance, health care personnel contacted surveillance staff to report suspect cases. In point- of-control surveillance, staff performed exhaustive screenings at key checkpoints in the area to detect and report suspect cases.

### CBS coverage

We restructured CBS to meet MoH standards and assure a manageable workload for CHW. One CHW covered a maximum of 35 households. CHW visited 15 households per day, with the 35 households covered every three days. Community leaders provided input on final numbers of CHW based on population estimates. Strong performing CHW or local leaders managed the CHW as supervisors. These supervisors replaced health center staff whose competing responsibilities precluded adequate supervision. Each supervisor managed a maximum of 10 CHW. CHW completed a two-day training in components of CBS. Supervisors completed one additional day of training.

### Community engagement

Community members contributed to surveillance activities from the start of the strengthening period. They participated in open meetings to provide discussion and feedback. With the phased roll-out, community members approved plans before activities proceeded. For example, names of CHW were not discussed until the number of CHW was approved. Training did not start until names were endorsed. The MoH guidelines required CHW to be residents of the neighborhood they covered, literate, vaccinated for EVD, in good standing with the community, and not employed in other EVD response activities. In the Mambasa CBS, we additionally asked the community to elect 50% women CHWs. The nominations were organized by the chiefs and other local leaders who provided the surveillance team with a final list of names. Supervisors were endorsed by the community, though selected by the surveillance team. Community leaders and supervisors who worked in the CBS acted as advocates, discussing the response with residents, and sharing feedback with the surveillance team.

### Tools and reporting

CHW submitted daily reports to supervisors who in turn submitted daily reports to the surveillance team. All tools were paper based for ease of reporting as partners lacked the devices and logistics required for electronic reporting. We revised existing tools to lighten reporting and added new tools to cover gaps. We aimed to have 95% of CHW reports daily. A small remuneration was paid to CHW and supervisors for each daily report submitted. In accordance with Riposte policy, CHW received approximately 5 USD per daily report submitted and supervisors 10 USD. The amount remained consistent over implementation. Remuneration provided appropriate compensation for time worked and motivated staff to submit reports. Daily reports included information on how many households CHW visited, in addition to number of people sensitized and sick. If no sick people were reported for the day (zero-reporting), CHW were still compensated for submitting a daily report.

The Riposte focused on compilation of a set of national, standardized indicators by health zone (Table 2. CBS Indicators). These were reported at daily coordination meetings. Centralized reporting meant data could not be disaggregated by smaller geographical units. The heavy workload left little time for longer-term analysis of trends. MSF focused on a subset of indicators which were analyzed weekly. Surveillance team staff and CHW supervisors held daily meetings to review alerts, provide coaching and discuss problems. Once a week, we held community meetings that were mandatory for CHW and open to community members.

**Table 2:** CBS Indicators

	Riposte Indicators	MSF Subset Indicators
**Household Visits**	Total Households Visited, Occupancy of men, women, <5	Total Households Visited
**Visitors** (Entries from other health areas)	Age, sex and provenance	-
**Travelers** (Departures from the health area)	Age, sex and destination	-
**Sick Persons**	Total and arrived for care	Total and arrived for care
**Alerts**	Investigated and validated	Investigated and validated
**Community Deaths**	Total and swabbed	Total and swabbed
**CHW performance**	Total alerts raised per CHW Percent of CHW reporting daily	Percent of CHW reporting daily

### Rural villages

We made considerable efforts to integrate rural villages into CBS. These areas were historically neglected in the response and were directly en route from areas of active transmission. Operational and budget limitations prevented proposed activities in these areas, such as mobile clinics and reimbursement of transport costs. However, to provide better support, the surveillance team began regular community meetings closer to the rural areas and increased supervisory visits.

### Health systems approach

The local health facility was converted to an ITC, which combined EVD testing and isolation with free PHC, referral services, and an ambulance network. Positive cases would be transferred from the ITC to the Ebola Treatment Center (ETC). Nurses triaged all patients arriving at the ITC. They received coaching in EVD case definitions and proper infection prevention and control during triage. Other alerts for diseases of epidemic potential were also reported during investigations, including malaria, acute respiratory infection (proxy for flu), measles, acute flaccid paralysis (proxy for poliomyelitis), typhoid fever, and yellow fever. Reporting on these diseases was integrated into the early warning and response system (EWARS) as recommended by WHO [11]. Health promotion teams comprised of local community members worked closely with the CHW and supported patients admitted to the ITC. They explained the disinfection and isolation process, discussed patient care, and liaised with patients’ family and friends.

## Results

### CBS coverage

The CBS system included 233 CHW: 115 in Binase health area covering 3,555 households and 118 Salama health area covering 4,139 households. The ratio of CHW to households decreased from 1:59 pre- strengthening to 1:33 post-strengthening (Table 3. *CBS Coverage*). A total of 27 supervisors managed CHW, reducing the ratio of supervisors to CHW from 1:14 pre-strengthening to 1:9 post-strengthening. The retention rate was 94.3%, with 247 CHW and supervisors completing training. CHW who resigned did so primarily because they found other paid positions. In rare cases, CHW were asked to leave due to violations of the code of conduct. In the first month of operations, 0% of CHW received timely payments, defined as payment within two weeks of work. By January 2019, 85% (n = 218) of CHW had received timely payments. Delays in the remaining 15% were related to logistical challenges with phone-based banking systems (M-Pesa) or human errors in payment details.

**Table 3:** CBS Coverage, Pre- and Post-System Strengthening

	Binase		Salama		Total	
	Pre	Post	Pre	Post	Pre	Post
**Total CHW**	65	115	65	118	130	233
**Ratio of CHW to Households**	1:53	1:31	1: 63	1:35	1:59	1:33
**Total Supervisors**	5	14	4	13	9	27
**Ratio of Supervisors to CHW**	1:13	1:8	1:16	1:9	1:14	1:9

### Community engagement

We held 32 feedback meetings with the community over 17 weeks. Four were preliminary meetings, 24 were weekly meetings and 4 were ad hoc meetings to address specific issues that arose. With the phased roll-out, community members reviewed and advised on activities before they started. Community recommendations improved system coverage and engagement. These included adding additional CHW in areas with difficult terrain or long distances, running separate weekly meetings for rural villages, and investing resources in PHC. The community respected guidelines for CHW selection. In Binase, 49.6% (n = 57) of the 115 CHW were women. In Salama, 50.8% (n = 60) of 118 CHW were women.

### CBS alerts and reporting

The system had consistently high reporting rates with an average of 97.4% (range: 75 − 100%) CHW reporting daily *(*Table 4. *CBS Reporting and Alerts*). Of the 6,711 sick persons identified, 98.1% (n = 6,583) were classified as alerts. The remaining 1.9% (n = 128) had medical conditions that clearly disqualified them as alerts, for example, elderly persons with chronic disease, no fever, and no change of symptoms in the past 2 weeks. Of the alerts, 97.4% (n = 6,410) were investigated to determine whether they met the suspect case definition. This is likely an overestimate, as alerts that were not investigated may have been excluded from the data. Of the alerts raised, 3.0% (n = 190) were validated for testing and isolation. Of the invalidated alerts referred for PHC, 73.1% (n = 4,905) arrived at the ITC.

**Table 4:** CBS Reporting and Alerts

	Binase^1^	Salama^2^	Total
**Daily Reports Submitted – % (n)**	96.3 (12,227)	99.6 (6,331)	97.4 (18,558)
**Sick People Referred (n)**	3,877	2,834	6,711
**Sick People Alerted - % (n)**	96.8 (3,752)	99.9 (2,831)	98.1 (6,583)
**Alerts Investigated - % (n)**	95.5 (3,583) ^1^	99.9 (2,827)	97.4 (6,410)
**Alerts Validated - % (n)**	2.7 (94) ^1^	3.4 (96)	3.0 (190)
**Referrals Arrived for PHC - % (n)**	58.3 (2,260)	93.3 (2,645)	73.1 (4,905)

^1^ Data from weeks 1–13, ^2^ Data from weeks 6–13

**Fig. 2:**
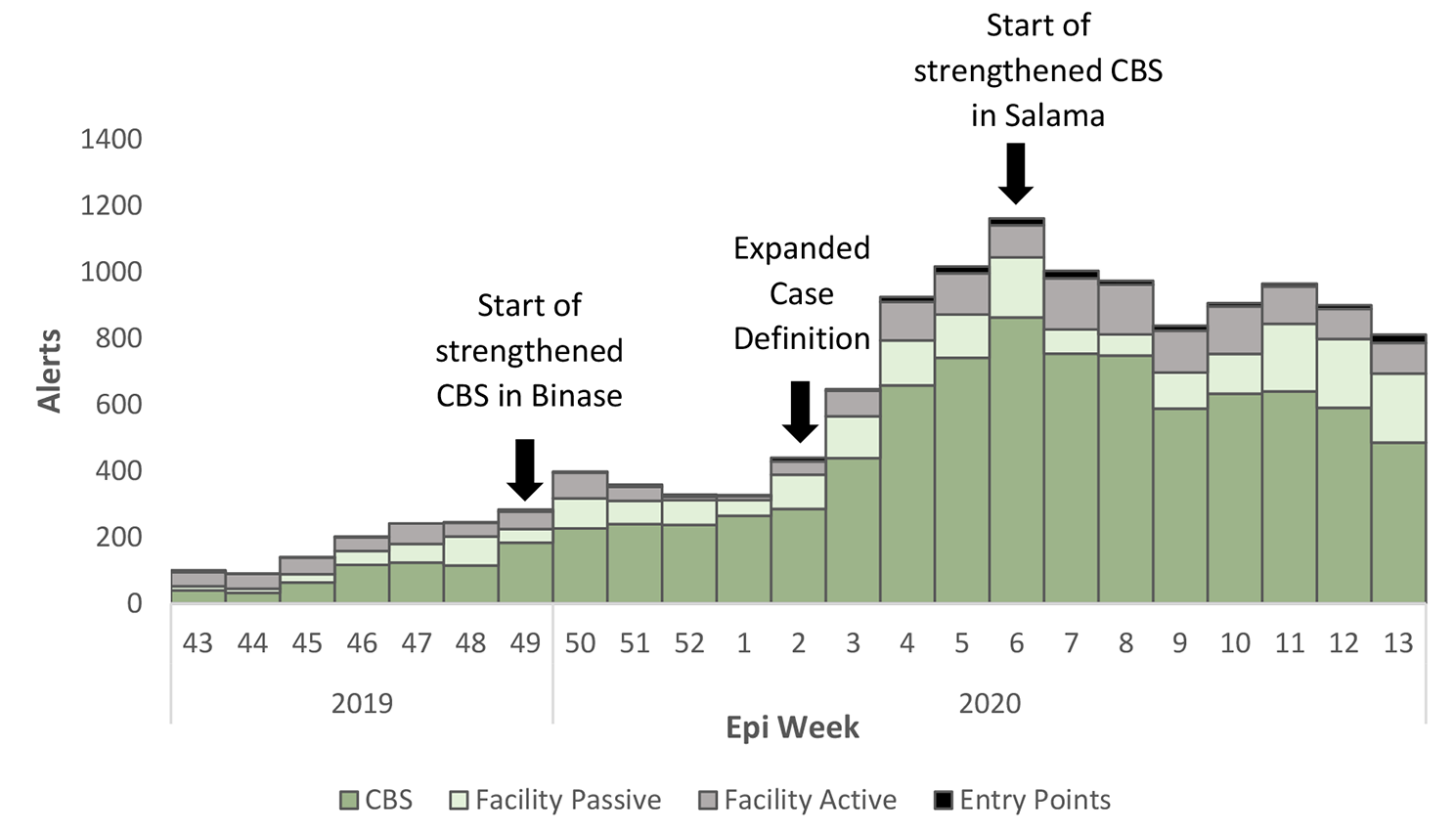
EVD Surveillance Alerts by Source

There was a notable increase in the contribution of CBS alerts to the surveillance system after strengthening activities and expansion of the case definition (Fig. 2. EVD Surveillance Alerts by Source). The CBS contribution increased from an average of 47.3% pre-strengthening to 69.0% post- strengthening. There was a decrease in proportion of alerts validated after the case definition was expanded. Prior to the expansion, an average of 18.9% of alerts from all sources were validated. After the expansion, the number fell to an average of 8.8%. The CBS contribution to death reporting declined from 69.4% pre- strengthening to 41.2% post- strengthening. Binase decreased from 57.1% pre- strengthening to 42.6% post and Salama decreased from 66.7% to 56.4%.

### Rural villages

The surveillance team had mixed results integrating the villages outside of the city limits into the system. In Binase health area, 12.8% of the population lived in rural villages and contributed 13.0% of alerts indicating adequate coverage by population. In Salama health area, 8.3% of the population lived in rural villages but contributed only 6.1% of the alerts indicating insufficient coverage.

## Discussion

### Coverage

The level of CBS coverage was necessary for an active EVD epidemic but would have been excessive in a non-emergency context. The community reported fatigue from the frequent household visits. It is possible that better trust between community members and response actors would have facilitated self-reporting, allowing a reduction in household visits. The increase in supervisors, transparent selection, and change in profile assured better efficiency and quality of alert investigation. It likewise improved supervision quality and enabled health center staff to focus on triage and patient care.

CHW maintained a high reporting rate throughout operations, consistently achieving > 90% CHW reporting daily. In a similar EVD CBS system in Sierra Leone, reporting rates of > 80% were considered high [18]. Our coverage area was notably smaller, with fewer CHW, fewer households and less frequent household visits. Incentives are perhaps the largest contributing factor to CHW retention [19]. While rates were fixed, timeliness of payments improved after system strengthening. Timely payments were recognized as a key motivator for individual and system level performance [20]. When CHW retention is high, staff can focus on improving the quality of the system. Tasks were divided between organizations. This improved capacity, but sometimes translated to ambiguous reporting lines and inconsistent decision-making.

### Community engagement

Lack of community engagement was a criticism and source of dangerous tension in much of the EVD response, as evidenced by the attacks on health care workers [21, 22]. The absence of community feedback, flawed recruitment of staff, and prioritization of EVD over other health needs created considerable tension with communities. A review of community engagement in COVID-19 emphasized early engagement with communities, continuous monitoring of activities, clear roles for actors, and open communication, as critical components of outbreak control [23].

Several factors helped build trust between actors. Community leaders were willing to put aside existing tension and work together. Ongoing collaboration, consultation, and routine meetings reinforced the partnership. Community recommendations greatly improved the quality of the CBS system. Engagement of community leaders, who at times were CHW and/or supervisors themselves, meant traditional hierarchies were integrated into the response. However, this may have also exacerbated existing imbalances in local power structures and promoted nepotism in CHW recruitment. CHWs had access to resources in the response, specifically paid employment, which at times created tension with those not employed in the response.

As women in the community are primarily responsible for the care of sick persons, their representation in CHW cohorts is important. While the community elected a good balance of men and women to the CHW cohort, the most active people during meetings were often men who held positions of power. Of note, we failed to integrate community members into leadership positions during implementation. A community advisory committee with diverse representation may have been a better way to facilitate engagement, assure more voices were heard, and create space for underrepresented groups. Despite weaknesses, the high retention rate of CHW, proportion of referrals who arrived for care, and number of alerts indicated a level of community acceptance of the system.

### Alerts

The change in contribution of CBS to total alerts may have been due in part to system strengthening, but also to the expansion in the EVD suspect case definition. The expanded case definitions were intended to improve early detection and decrease the possibility of missing a case. In practice, the quality of alerts and capacity to investigate them declined after expansion of the case definitions. In addition, they were highly sensitive but not specific enough to have clinical significance. When every sick person required investigation, many patients were erroneously classified as suspect cases. Previous analyses have shown that performance of an EVD case definition is dependent on community trust [24]. The sensitive case definitions may have contributed to unnecessary admissions to the ITC for testing and isolation. This likely hindered trust, resulting in community disengagement, delayed access to care, and system inefficiency.

The expansion in case definition also had serious operational implications. Surveillance workload increased and laboratory and isolation capacities were overwhelmed. ITC admissions doubled during this period [25]. Due to limited capacity, up to half of patients arriving for isolation and testing at the ITC had to be transferred to other facilities because of lack of space. The ITC was eventually compelled to expand capacity to provide space for more admissions. ITC staff also described being overburdened with the urgency and number of patients, reporting internally that quality of care was compromised. The CBS referral protocol, whereby all sick patients were referred to the ITC, meant most investigations were done at triage which compensated for the increase in alerts. Laboratory and isolation capacities, however, remained insufficient.

### Reporting

Despite efforts to lighten the burden of reporting, it remained complex, with numerous tools and indicators, arbitrary targets, inconsistent data quality and little actionable analysis. Importantly, reporting was challenging for CHW to manage, and little feedback was provided to the community. Indicator performance and reporting frequency were prioritized over data usefulness. The Riposte analysis cell posited a target of 27.2 alerts per 10,000 people per week with 20–60% of alerts validated [26]. The EVD Response Plan stressed the importance of CBS and aimed to have 50% of alerts originate from the community^8^. While targets may have originally been intended to have flexibility and be adapted to the context, in practice they were interpreted rigidly.

There was little formal documentation of the targets despite their strong emphasis in daily coordination meetings. Surveillance team members were frequently chastised in these meetings if daily targets were not met. In general, targets were attained. However, many indicators became models of Goodhart’s law that once “a measure becomes a target, it ceases to be a good measure” [27]. One example was the pressure to focus on quantity over quality of alerts. Teams may have intentionally or unintentionally influenced data to achieve targets. The division of tasks between organizations helped with heavy reporting requirements, enabling team members to spend time on analysis which informed operations.

In future responses, targets should be used with caution, interpreted in the broader scope of CBS performance, grounded in evidence, and adapted to the context. Data collection could be limited to variables that will be (1) analyzed in a timely manner and (2) used to improve the response [28]. While some indicators were reported to the community during open weekly meetings, a more formal community feedback mechanism is recommended. A review of requirements early in the response may have reduced the reporting burden. Community reporting of deaths declined after CBS implementation. We attribute the decline in community reporting of deaths to the strong referral system. Most sick persons arrived for care at the ITC, severe cases were transferred to the hospital for care and deaths may have been registered as facility instead of community deaths. It is also possible that early detection of disease and prompt treatment, as well as enhanced free PHC could have contributed to an overall reduction in mortality in the communities, though we lack data to demonstrate it.

### Rural villages

Surveillance in rural areas outside Mambasa posed challenges. Insecurity and distance made it difficult for CHW and community members in those areas to come to town, for surveillance team members to conduct investigations, and for sick persons to get care. The limited options for PHC contributed to tension, as community members perceived the response to prioritize EVD response over PHC. In Binase, our efforts to improve coverage and services in rural areas improved over time. In Salama, activities did not achieve the same results, due to the late start-up and shorter implementation period. In future responses, dedicated staff could be recruited to oversee CBS in rural areas.

### Health systems approach

The Riposte sought to embed EVD activities within the existing health system and emphasized the need for free healthcare as a pre-requisite for EVD control [8]. The PHC services, referral system, and ambulance network integrated EVD response activities to the existing health system and facilitated CBS performance. More importantly, combined with the extremely sensitive case definition which alerted all persons sick with any disease, the PHC, referral system and ambulance network provided a continuum of care that addressed community prioritized health needs. These included care for common pathologies such as malaria, diarrhea, and respiratory infections [29]. In comparison to these, EVD affected a small percentage of the population [30]. When community priorities were addressed, EVD activities were better accepted. While the medical teams worked diligently to provide quality PHC services, the testing and isolation unit consumed most of staff time and project resources. Future responses could provide a better balance between EVD testing and isolation and PHC.

Persuading patients to isolate and be tested in the ITC was another ongoing challenge. The efforts of health promotion teams did much to alleviate concerns. These teams worked consistently to help community members understand why isolation and testing were necessary to preserve the health and safety of their community. As the health promoters themselves came from the community, there was more inherent trust in their communication and more weight to their explanations. Patients had to receive two consecutive negative test results to be discharged from the isolation unit. The health promotion teams were likewise indispensable in communicating the results to patients, discharging them, and helping to defend against stigma once they re-entered the community. Finally, the uncertainty of the epidemic meant that the project exit was rushed and inadequately planned. As the CBS was adapted from the existing MoH system, a clear exit strategy at the end of the response would have allowed the CBS to be reintegrated into the health system, potentially focusing on other infectious diseases, with redefined roles and sufficient time for handover.

### Limitations

There was no EVD transmission in the health areas over the implementation period, meaning we cannot draw conclusions on how the CBS would have performed under active transmission. A proper evaluation of CBS would have assessed its main attributes: simplicity, flexibility, acceptability, sensitivity, positive predictive value, representativeness, and timeliness [31]. The EVD response was marked by poor information management with fragmented and incomplete databases [8]. This makes CHW contribution difficult to quantify and precludes a more complete evaluation of the system. Data quality was a point for improvement and emphasis on meeting targets meant system performance may have been overestimated. Finally, EWARS data reported through the MoH health system was unavailable for analysis which prevented the evaluation of CBS performance for other diseases of epidemic potential.

## Conclusion

Previous studies have shown that CBS performance is mediated by operational demands for supervision, capacity to investigate alerts, and integration of the system into routine reporting [11]. By strengthening the EVD CBS system, we aimed to address these factors, along with the historic lack of community engagement, inadequate coverage and competing health priorities. The DRC EVD system demonstrated the capacity of CBS to provide both early disease detection and improved access to healthcare during an emergency response. Early and consistent community involvement is vital to the success of CBS. It was a critical factor in system performance, local acceptance of EVD activities, and health service provision. Our experience shows the potential for improved results when actors work together, and key structural components of CBS are supported. We provide a summary of the operational lessons learned that could inform CBS in similar emergency response contexts (Table 5).

**Table 5:** EVD CBS Lessons Learned

Area	Lessons Learned
Coverage	• The ratio of 1:35 CHW to households and 1:15 CHW to household visits per day assured equal and adequate coverage. The level was appropriate for an active EVD epidemic but would be excessive outside of an emergency and resulted in CHW and household fatigue. • The ratio of 1:10 supervisors to CHW was a manageable workload for staff while assuring adequate supervision and high reporting rates. • The increase in supervisors, transparent selection, and change in profile improved alert investigation and supervision quality, and enabled health center staff to focus on triage and patient care. Multiple partners meant management positions were inflated. This had benefits in division of tasks but translated to ambiguous reporting lines and inconsistent decision-making.
Community Engagement	• Early and consistent community involvement proved vital to the success of the system, reinforcing system performance, local acceptance of activities, and health service provision. • Community feedback, including recommendations to add additional CHW, run separate meetings in rural villages, and strengthen PHC services, improved system coverage and performance. • Phased roll-out of activities ensured time for community discussion and approval. • Engaging local chiefs and other community leaders as supervisors and CHW meant traditional hierarchies were integrated into the response • Leadership came from outside of the community. Future response should ensure there is community representation in senior, decision-making positions. • Engagement with local chiefs may have exacerbated existing imbalances in local power structures. A community advisory committee with diverse representation may have been a better way to facilitate engagement, assure more voices were heard, and create space for underrepresented groups.
Rural Villages	• Additional time and resources were needed to accommodate rural villages. • Holding separate community meetings close to rural villages and increasing visits provided better support to the CHW and improved system performance in these areas. • Increasing access to PHC services in rural areas would have increased capacity for early disease detection and assured more sick persons received care. • If resources allow, dedicated surveillance staff should be recruited to oversee CBS in rural areas.
Reporting	• Data collection should be limited to information that (1) is useful to the response and (2) can be analyzed in a timely manner. A review of reporting requirements early in the response may have mitigated reporting challenges. • Targets should be used with caution, interpreted in the broader scope of CBS performance, grounded in evidence and adapted to the specific context in which they are used. • The partnership between organizations was useful under the heavy reporting requirements, enabling team members to spend time on analysis which informed operations.
Case Definition	• The expanded case definitions were highly sensitive but not specific enough to have clinical significance. The change overwhelmed response efforts in surveillance, and laboratory and isolation capacity. • If a more sensitive case definition is needed, actors should ensure there is increased operational capacity for alert investigations, testing, and isolation.
Health System Approach	• In addition to providing a continuum of care, the free PHC services, referral system, and ambulance network addressed community-prioritized health needs. When community priorities were addressed, EVD activities were better accepted. • Efforts could have been made to invest more in the PHC component. The EVD testing and isolation unit took priority, disproportionately consuming staff time and project resources. • The support of the locally recruited health promotion team helped greatly with communication and alleviation of concerns around ITC admission. • A clear exit strategy would have allowed the CBS to be reintegrated into the health system, focusing on other infectious diseases, with redefined roles.

## Data Availability

The datasets generated during and/or analyzed during the current study are not publicly available due to MSF policy on data protection. MSF has a managed access system for data sharing. Data are available on request in accordance with MSF’s data sharing policy. Requests for access to data should be made to data.sharing@msf.org.
